# GRAPE: graph-regularized protein language modeling unlocks TCR-epitope binding specificity

**DOI:** 10.1093/bib/bbaf522

**Published:** 2025-10-06

**Authors:** Xiangzheng Fu, Li Peng, Haowen Chen, Mingqiang Rong, Yifan Chen, Dongsheng Cao, Sisi Yuan, Aiping Lu

**Affiliations:** Institute of Artificial Intelligence Application, College of Computer and Information Engineering, Central South University of Forestry and Technology, No. 498 Shaoshan South Road, Tianxin District, Changsha, Hunan 410004, China; School of Chinese Medicine, Hong Kong Baptist University, 15 Baptist University Road, Kowloon Tong, Kowloon, Hong Kong SAR 999077, China; College of Computer Science and Engineering, Hunan University of Science and Technology, No. 1 Taoyuan Road, Yuhu District, Xiangtan, Hunan 411201, China; College of Science and Electronic Engineering, Hunan University, 2 Lushan South Road, Yuelu District, Changsha, Hunan 410082, China; The National & Local Joint Engineering Laboratory of Animal Peptide Drug Development, College of Life Sciences, Hunan Normal University, 36 Lushan Road, Yuelu District, Changsha, Hunan 410081, China; Institute of Artificial Intelligence Application, College of Computer and Information Engineering, Central South University of Forestry and Technology, No. 498 Shaoshan South Road, Tianxin District, Changsha, Hunan 410004, China; School of Information Engineering, Changsha Medical University, No. 1501 Leifeng Road, Wangcheng District, Changsha, 410219, China; Xiangya School of Pharmaceutical Sciences, Central South University, No. 172 Tongzipo Road, Yuelu District, Changsha, Hunan 410003, China; Department of Bioinformatics and Genomics, The University of North Carolina at Charlotte, 9201 University City Blvd, Charlotte, NC 28223, United States; School of Chinese Medicine, Hong Kong Baptist University, 15 Baptist University Road, Kowloon Tong, Kowloon, Hong Kong SAR 999077, China

**Keywords:** TCR-epitope binding, protein language models, graph regularization, AUC-maximization

## Abstract

T-cell receptor (TCR)-epitope binding prediction is critical for immunotherapies but remains challenged by sparse interaction networks and severe class imbalance in training data. Current graph neural network (GNN) approaches for predicting TCR-epitope binding (TEB) fail to address two key limitations: over-smoothing during message propagation in sparse TCR-epitope graphs and biased predictions toward dominant epitope-TCR pairs. Here, we present GRAPE (Graph-Regularized Attentive Protein Embeddings), a framework unifying spectral graph regularization and imbalance-aware learning. GRAPE first leverages protein language models (ESM-2) to generate evolutionary-informed TCR/epitope embeddings, constructing a topology-aware interaction graph. To mitigate over-smoothing, we introduce spectral graph regularization, explicitly constraining node feature smoothness to preserve discriminative patterns in sparse neighborhoods. Simultaneously, a dynamic edge reweighting module prioritizes unobserved TCR-epitope edges during graph propagation, coupled with a differentiable area under the ROC curve-maximization objective that directly optimizes for imbalance resilience. Extensive benchmarking on public datasets demonstrates that GRAPE significantly outperforms state-of-the-art methods in TEB prediction. This work establishes GRAPE as a robust framework for elucidating TCR-epitope interactions, with broad applications in immunology research and therapeutic design.

## Introduction

T-cells play a central role in adaptive immunity by recognizing epitopes presented by major histocompatibility complex (MHC) molecules, leading to T-cell activation and the initiation of immune responses against pathogens, tumor cells, and viruses [1–3]. In tumor immunology, short peptide neoantigens presented by MHC molecules typically originate from somatic mutations and are critical for T-cell recognition and immune activation A detailed analysis of the TCR-epitope binding (TEB) mechanism is essential for advancing cancer immunology, identifying autoimmune antigens, and designing effective vaccines [4]. However, the complexity of this recognition process makes experimental validation of TCR-epitope interactions time-consuming and costly [5]. Therefore, various computational approaches have been developed to simulate TCR-epitope interactions efficiently [6, 7].

In adaptive immune responses, peptides, commonly referred to as epitopes, are presented by MHC molecules on the cell surface and subsequently recognized by TCRs. While TCRs bind both the epitope and the corresponding MHC molecule, the core binding region primarily involves the complementarity-determining region 3 of the TCR β-chain (CDR3β) and the epitope [1]. Recent advances in high-throughput tetramer TCR sequencing, tetramer analysis [8], and T-cell scanning [9] have facilitated the large-scale generation of TCR–epitope binding data, now stored in public databases such as VDJdb [10], IEDB [11], and McPAS-TCR [12]. However, despite these expanding datasets, the available data remain limited compared to the immense theoretical diversity of TCRs. Additionally, since a single epitope can bind multiple TCRs, the data distribution is highly imbalanced, further complicating computational predictions. The scarcity and imbalance of data present significant challenges for developing accurate and efficient computational models.

In response to these challenges, machine learning-based computational methods have been widely adopted in recent years. Early research focused on constructing epitope-specific models to identify TCR patterns associated with particular epitopes. These methods evolved from simple sequence alignment to advanced machine learning models, such as Random Forest (e.g. TCRex [13]) and Gaussian Process Classifiers [14]. However, it became evident that training separate models for each epitope requires extensive epitope-specific TCR data, which is often difficult to obtain. To address this limitation, researchers have shifted toward developing more generalized models capable of predicting the binding specificity between any TCR and peptide–MHC (pMHC) epitope. Several recent models have advanced this approach. TEINet [15], a deep learning framework leveraging transfer learning, independently pretrains encoders to embed TCR and epitope sequences into high-dimensional numerical representations, enabling accurate binding specificity prediction. TEIM [16] leverages large-scale sequence-level data for pretraining and captures key residue spatial interactions during fine-tuning, addressing the scarcity of residue-level data while significantly improving predictive accuracy and generalization. pMTnet [17] employs a phased training strategy and embedded feature extraction to convert the high-dimensional complexity of TCR-pMHC binding into numerical representations, addressing data scarcity, and imbalance. More recently, GTE [18] has introduced graph topology structures, leveraging heterogeneous graph neural networks (GNNs) to model complex TCR-epitope interactions, demonstrating strong predictive performance.

Despite the advancements in TEB prediction, existing methods still face several challenges. First, these models rely on specific datasets to extract sequential representations, and the limited availability of data restricts their generalization capabilities. Second, deep learning approaches, particularly GNNs, are prone to over-smoothing in sparse neighborhoods and struggle to effectively model global dependencies. To address these challenges, we proposed a novel TEB prediction model that integrates protein language models with graph regularization techniques to enhance TCR-epitope interaction predictions. Our contributions can be summarized as follows:

We developed GRAPE, a novel TEB prediction model that integrates protein language models with graph regularization techniques to capture both local graph features and global dependencies while mitigating over-smoothing in sparse neighborhoods.

We implemented a dynamic graph learning strategy together with an area under the ROC curve (AUC)-maximization approach to effectively address data imbalance during training, ensuring robust model performance across diverse datasets.

Through large-scale benchmarking, GRAPE achieves state-of-the-art performance across diverse TEB prediction tasks, demonstrating its potential to accelerate therapeutic discovery in cancer immunology and vaccine design.

## Materials and methods

### Data preparation

This study utilized four datasets collected by previous research to evaluate the performance of the proposed and comparison models, as shown in Table 1. TEINet dataset [15] contains 41 610 TCRs, 180 epitopes, and 44 682 known TEBs. pMTnet dataset [17] applies quality indicators from the source databases to retain only high-confidence TCR-epitope pairs [10, 12, 19, 20]. For instance, for VDJdb and TCRGP [7], only records with a vdj.score >0 are included to enhance reliability. The final filtered dataset comprises 31 203 TEBs, involving 28 604 TCRs and 426 epitopes. VDJdb dataset [10] initially includes 89 847 curated CDR3 sequences covering TCR ɑ and β chains across human, monkey, and mouse species. After filtering, only human-derived data were considered, resulting in a refined dataset of 38 533 unique CDR3-epitope pairs, including 36 033 TCRs and 1002 epitopes. McPAS-TCR Dataset [12] is a manually curated repository of pathology-associated TCR sequences, comprising 40 033 TEBs from both mouse and human species. After filtering, the final dataset includes 5102 unique CDR3-epitope pairs, with 4975 TCRs associated with 244 epitopes.

**Table 1:** Statistical details of the TEINet, pMTnet, VDJdb, and McPAS datasets

Dataset	TCRs	Epitopes	Pairs
TEINet	41 610	180	44 682
pMTnet	28 604	426	31 203
VDJdb	36 033	1002	38 533
McPAS	4975	244	5102

### Method

This study proposes GRAPE, a model that integrates protein language models and graph regularization techniques to predict TEB specificity. First, we leveraged the rich information in the ESM model to generate TCR and epitope representations. These representations, combined with known TEBs, constructed a TCR-epitope graph. Next, we designed an encoder incorporating attention mechanisms and graph regularization to capture both local and global features while mitigating over-smoothing in sparse neighborhoods. Additionally, we employed a dynamic graph learning strategy and AUC-maximization techniques to address class imbalance in the TCR-epitope datasets.

### Model architecture

**Figure 1:**
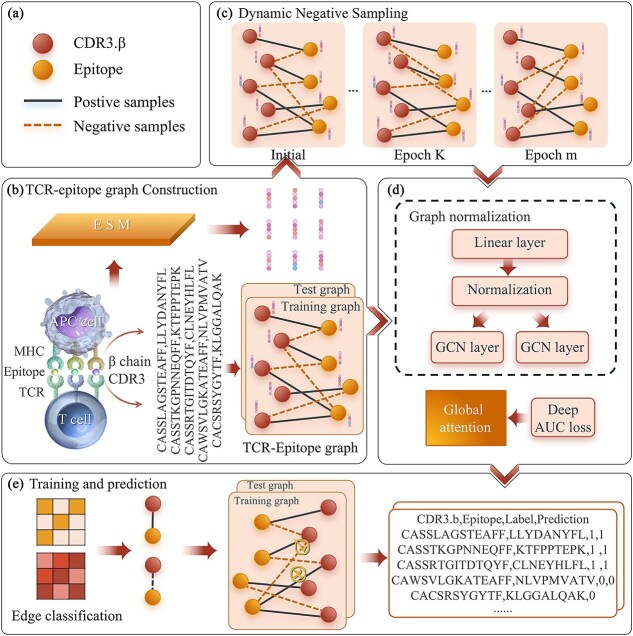
GRAPE model’s architecture, comprising: (a) data symbols, (b) construction of a TCR-epitope bipartite graph, (c) dynamic negative sampling, (d) graph-regularized attention encoder and AUC maximization, and (e) training and prediction.

Fig. 1 depicts the GRAPE model’s architecture, which encompasses: (A) data symbols, (B) construction of a TCR-epitope bipartite graph, (C) dynamic sampling, (D) graph-regularized attention encoder and AUC-maximization, and (E) prediction. Module (A) indicates the meanings of some symbols. In module (B), the ESM2.0 model is used to generate embeddings for TCR and epitope sequences, which are then used to construct a TCR-epitope bipartite graph. In module (C), dynamic sampling techniques adaptively update negative TCR-epitope pairs. In module (D), a graph-regularized attention encoder extracts topological features of TCRs and epitopes. And an AUC loss function was employed to address potential data imbalance during training. Finally, in module (E), the representations of TCR-epitope pairs are concatenated, and scores are calculated through a multilayer perceptron (MLP).

### Protein language model

The limited quantity of TCR and epitope data, combined with the short length of their sequences, presents a challenge in accurately characterizing these sequences. ESM2.0, an advanced protein language model, is designed to extract features from large-scale protein sequence data. It utilizes a Transformer architecture and Masked Language Modeling (MLM) techniques, where the model predicts masked amino acid fragments by randomly concealing segments within the sequence. This approach allows the model to capture hidden semantic and structural information within the sequence.

In recent years, transfer learning has increasingly been applied to generate embeddings for TCRs and epitopes [15; 16]. However, existing model frameworks have not been specifically optimized for large-scale protein language models. As a result, when using embeddings extracted by ESM2.0, their predictive performance often lags behind that of data-driven models trained on specific TCR or epitope sequences (e.g. TCRpeg). To address this, we designed a dedicated model architecture tailored to better leverage the embeddings generated by ESM2.0. In our approach, the ESM2.0 is used to extract deep semantic features from TCR and epitope sequences. By inputting these sequences into the pretrained ESM2.0, we generate high-dimensional feature vectors, which exhibited excellent applicability for subsequent interaction prediction tasks. Notably, our experiments revealed that smaller parameter settings often yield optimal performance. This suggests that the capacity of smaller models is sufficient to capture the key features of the data, while larger models may introduce redundant features, which can negatively impact performance.

### T-cell receptor-epitope bipartite graph construction

The physical interaction network between proteins is essential for information transmission and functional coordination in biological systems [21–23]. However, previous methods have primarily focused on learning sample embeddings and distance metrics in feature space, often neglecting the rich information encoded in network topology. This underutilization of topological features may constrain the ability of models to accurately represent complex biological mechanisms. In the protein–protein interaction (PPI) networks, all nodes in a homogeneous graph are considered identical [23]. However, due to their distinct biological properties, TCRs and epitopes should be treated as different node types. We construct a heterogeneous graph ***G* = *(V,E,X)***, where ***V*** represents the set of nodes, including TCR and epitope nodes. The edge set ***E*** defines relationships between these nodes, with the number of edges corresponding to the total number of positive and negative TCR-epitope pairs. Edges are labeled as 1 for positive interactions and 0 for negative interactions. To maintain structural consistency, we use a single edge type to represent both positive and negative samples, framing the task as an edge classification task.

The feature matrix ***X*** contains node representations, where each node ***v_i_*** is associated with a feature vector ***x_i_*** extracted from a pre rained ESM2.0: ***x_i_* = *ESM2(v_i_)***. In the constructed graph, an edge ***e_i_***_***j***_ represents the connection between nodes ***v_i_*** and ***v***_***j***_, with the corresponding label ***y_i_***_***j***_ indicating the presence (1) or absence (0) of a binding interaction. Thus, the edge set ***E*** and the label set ***Y*** can be formally expressed as follows:


(1) \begin{equation*} \boldsymbol{E}=\left\{\left({\boldsymbol{v}}_{\boldsymbol{i}},{\boldsymbol{v}}_{\boldsymbol{j}}\right)\ |\ {\boldsymbol{v}}_{\boldsymbol{i}}\in \boldsymbol{V},{\boldsymbol{v}}_{\boldsymbol{j}}\in \boldsymbol{V}\right\},\boldsymbol{Y}=\left\{{\boldsymbol{y}}_{\boldsymbol{i}\boldsymbol{j}}\ |\ {\boldsymbol{e}}_{\boldsymbol{i}\boldsymbol{j}}\in \boldsymbol{E}\right\} \end{equation*}

### Graph regularization-attention encoder

GNNs are widely used for analyzing network data due to their ability to capture topological structures. However, conventional GNNs face two challenges in TEB prediction. First, TCRs or epitopes with sparse neighbors may lead to over-smoothing during message propagation, reducing predictive reliability. Second, traditional GNNs primarily rely on local neighborhood information, limiting their ability to capture global dependencies. Then we propose an encoder that integrates graph regularization with global attention mechanisms.

### Graph regularization technique

Sparse neighborhoods in graphs can lead to over-smoothing during message propagation, a challenge observed in previous studies [24]. This issue is also present in the TCR-epitope bipartite graph. To mitigate this effect, we incorporate graph regularization techniques, inspired by prior work on variational graph autoencoders [25, 26]. Given a feature matrix $\boldsymbol{X}={\left[\overrightarrow{{\boldsymbol{x}}_{\mathbf{1}}},\overrightarrow{{\boldsymbol{x}}_{\mathbf{2}}},\boldsymbol{\cdots},\overrightarrow{{\boldsymbol{x}}_{\boldsymbol{n}}}\right]}^{\boldsymbol{T}}$, where ${\boldsymbol{x}}_{\boldsymbol{i}}\boldsymbol{\in}{\boldsymbol{R}}^{\boldsymbol{m}}$ represents the content feature vector of node ***v***_***i***_ and ***n*** is the total number of nodes. The Graph Normalized Convolutional Network (GNCN) first generates a transformed feature vector $\overrightarrow{\boldsymbol{h}}\boldsymbol{\in}{\boldsymbol{R}}^{\boldsymbol{f}}$ using a learnable weight matrix $\boldsymbol{W}\boldsymbol{\in }{\boldsymbol{R}}^{\boldsymbol{m}\times \boldsymbol{f}}$:


(2) \begin{equation*} \overrightarrow{{\boldsymbol{h}}_{\boldsymbol{i}}}=\overrightarrow{{\boldsymbol{x}}_{\boldsymbol{i}}}\boldsymbol{W} \end{equation*}

We employ the GNCN to generate a normalized feature transformation vector $\overrightarrow{\boldsymbol{n}}\boldsymbol{\in}{\boldsymbol{R}}^{\boldsymbol{f}}$, which is then propagated to generate the node embedding $\overrightarrow{\boldsymbol{z}}\boldsymbol{\in}{\boldsymbol{R}}^{\boldsymbol{f}}$. Based on the regularized representation, GNCN performs message propagation and outputs the embedding of node ***i***:


(3) \begin{equation*} \overrightarrow{{\boldsymbol{z}}_{\boldsymbol{i}}}=\frac{\mathbf{1}}{{\boldsymbol{d}}_{\boldsymbol{i}}+\mathbf{1}}\overrightarrow{{\boldsymbol{n}}_{\boldsymbol{i}}}+\sum_{\boldsymbol{j}\in{\boldsymbol{N}}_{\left(\boldsymbol{i}\right)}}\frac{\mathbf{1}}{\sqrt{{\boldsymbol{d}}_{\boldsymbol{i}}+\mathbf{1}}\sqrt{{\boldsymbol{d}}_{\boldsymbol{j}}+\mathbf{1}}}\overrightarrow{{\boldsymbol{n}}_{\boldsymbol{j}}} \end{equation*}

where $\overrightarrow{{\boldsymbol{n}}_{\boldsymbol{i}}}=\boldsymbol{s}\frac{\overrightarrow{{\boldsymbol{h}}_{\boldsymbol{i}}}}{\left\Vert \overrightarrow{{\boldsymbol{h}}_{\boldsymbol{i}}}\right\Vert }$, $\boldsymbol{s}\boldsymbol{\in}\boldsymbol{R}$ is the scaling parameter, be a scaling constant, representing the norm of the hidden features being propagated. For the feature matrix $\boldsymbol{X}\boldsymbol{\in }{\boldsymbol{R}}^{\boldsymbol{m}\times \boldsymbol{f}}$ and the adjacency matrix ***A***, the GNCN is defined as follows:


(4) \begin{equation*} \boldsymbol{GNCN}\left(\boldsymbol{X},\boldsymbol{A},\boldsymbol{s}\right)=\boldsymbol{s}{\hat{\boldsymbol{D}}}^{-\frac{\mathbf{1}}{\mathbf{2}}}\hat{\boldsymbol{A}}{\hat{\boldsymbol{D}}}^{-\frac{\mathbf{1}}{\mathbf{2}}}\boldsymbol{g}\left(\boldsymbol{X}\boldsymbol{W}\right) \end{equation*}

where $\boldsymbol{g}\left({\left[\overrightarrow{{\boldsymbol{h}}_{\mathbf{1}}},\overrightarrow{{\boldsymbol{h}}_{\mathbf{2}}},\cdots, \overrightarrow{{\boldsymbol{h}}_{\boldsymbol{n}}}\right]}^{\boldsymbol{T}}\right)={\left[\frac{\overrightarrow{{\boldsymbol{h}}_{\mathbf{1}}}}{\left\Vert \overrightarrow{{\boldsymbol{h}}_{\mathbf{1}}}\right\Vert },\frac{\overrightarrow{{\boldsymbol{h}}_{\mathbf{2}}}}{\left\Vert \overrightarrow{{\boldsymbol{h}}_{\mathbf{2}}}\right\Vert },\cdots, \frac{\overrightarrow{{\boldsymbol{h}}_{\boldsymbol{n}}}}{\left\Vert \overrightarrow{{\boldsymbol{h}}_{\boldsymbol{n}}}\right\Vert}\right]}^{\boldsymbol{T}}$represents the node embedding matrix, ***W*** is a trainable weight matrix, $\hat{\boldsymbol{D}}$ is the degree matrix of $\hat{\boldsymbol{A}}$, and ***s*** is a scaling constant. GNCN integrates both structural information (from the adjacency matrix) and content information (from the feature matrix) by leveraging both the adjacency and feature matrices. By incorporating normalization and the scaling constant ***s***, the model prevents the feature values from becoming excessively amplified or diminished during propagation, thus preserving semantic information. This effectively mitigates the over-smoothing issue, enabling accurate modeling of local relationships between neighboring nodes.

### Global attention mechanism

In this study, a global attention mechanism is introduced to model and optimize the global dependencies between nodes. The key idea is to allow any node in the graph to dynamically attend to other nodes, thereby capturing global dependencies effectively. Specifically, in the TCR-epitope bipartite graph ***G = (V,E,X)***, the initial feature vector ***x***_***i***_ (extracted using ESM2.0) is updated through the following attention mechanism:


(5) \begin{equation*} \boldsymbol{Attn}\left(\boldsymbol{H}\right)=\boldsymbol{Softmax}\left(\frac{\boldsymbol{Q}{\boldsymbol{K}}^{\boldsymbol{T}}}{\sqrt{\boldsymbol{h}}}\right)\boldsymbol{V},\boldsymbol{Q}=\boldsymbol{H}{\boldsymbol{W}}_{\boldsymbol{Q}},\boldsymbol{K}=\boldsymbol{H}{\boldsymbol{W}}_{\boldsymbol{K}},\boldsymbol{V}=\boldsymbol{H}{\boldsymbol{W}}_{\boldsymbol{V}} \end{equation*}

where $\boldsymbol{H}\boldsymbol{\in }{\boldsymbol{R}}^{\boldsymbol{m}\times \boldsymbol{f}}$ represents the hidden representation matrix, ***h*** denotes the dimension of the hidden representation, and ${\boldsymbol{W}}_{\boldsymbol{Q}}{\boldsymbol{W}}_{\boldsymbol{K}}{\boldsymbol{W}}_{\boldsymbol{V}}\boldsymbol{\in}{\boldsymbol{R}}^{\boldsymbol{m}\times \boldsymbol{f}}$ are the trainable weight matrices of the linear projection layer. The attention score matrix ${\mathbf{A}}^{\prime }=\mathbf{Softmax}\left(\frac{\mathbf{Q}{\mathbf{K}}^{\mathbf{T}}}{\sqrt{\mathbf{h}}}\right)$ records the attention weights between all node pairs, where ***α*** represents the attention score between nodes ***u*** and ***v***. Finally, by multiplying ***A′*** with the node representation matrix ***V***, the model globally updates node features, effectively capturing contextual semantic information and global dependencies.

In TEB prediction, the task’s heterogeneity and complex biological interactions prevent local topological features from fully capturing semantic associations. Therefore, a global attention mechanism enhances the model’s discriminative ability and predictive performance by dynamically integrating global TCR-epitope dependencies.

### Encoder

We propose a novel encoder that integrates graph regularization and a global attention mechanism to accurately extract local and global information from the TCR-epitope bipartite graph. Graph regularization enhances the modeling of TCR-epitope interactions by extracting fine-grained topological information while mitigating over-smoothing in sparse neighborhoods, leading to more robust TCR and epitope representations. Meanwhile, the global attention mechanism captures nonlocal dependencies at the sequence level, enriching global contextual semantics. By integrating local topological structures with global semantic information, the encoder significantly improves feature representation and predictive performance. Additionally, the model partially mitigates heterogeneity by using separate embedding channels for TCRs and epitopes, combined with attention-based encoders.

### Dynamic negative sampling strategy

During the construction of the TCR-epitope bipartite graph, identifying high-quality negative TCR-epitope pairs is challenging due to the limited number of known interactions and the abundance of unobserved pairs. Most existing methods directly classify unobserved TCR-epitope pairs as negative samples, which can result in misclassification of unknown interactions. To mitigate this issue, we adopt a dynamic negative sampling strategy, inspired by previous work [18], to generate a diverse set of negative samples and improve model robustness.

In the early training stages, unknown TCR-epitope pairs are randomly selected as negative samples and added to the initial training graph, which initially contains only positive samples. For each epitope, a predefined number of TCRs (default: 10) are randomly chosen to generate negative samples. To prevent overlap with positive samples, the selection process ensures their distinction. If conflicts arise, remaining negative samples are prioritized for inclusion.

During training, negative samples are dynamically updated based on each iteration’s prediction results. Specifically, misclassified negative samples are retained, while correctly predicted ones are resampled at a specified ratio (M%). Resampling follows the same approach as the initial sample collection. To ensure effective learning, the model is evaluated after each update. If performance surpasses the previous best, dynamic resampling continues in the next epoch. As shown in Fig. 1, light-colored edges indicate updated negative samples. This strategy exposes the model to a diverse set of negative samples with minimal cost, significantly improving training efficiency.

### Deep AUC-maximization

The number of observed TEB data is typically limited. To enhance the model’s learning ability and utilize larger datasets, a common approach is to introduce additional negative samples [26]. However, this can lead to data imbalance, which may be further exacerbated by dynamic negative sampling strategies. To evaluate model performance, the AUC is widely used, as it quantifies the model’s ability to rank positive samples higher than negative ones. Given the impact of data imbalance, loss functions designed to optimize AUC not only help mitigate this issue but also significantly improve predictive performance [27].

Consider the input space $\boldsymbol{X}\boldsymbol{\in}\boldsymbol{R}$ and output space $\boldsymbol{Y}=\left\{-\mathbf{1},\mathbf{1}\right\}$, where training data is drawn from an unknown distribution ***P*** and satisfies the independent and identically distributed (i.i.d.) assumption, such that ***Z* = *X* × *Y***. The ROC curve is constructed based on the relationship between the true positive rate (TPR) and the false positive rate (FPR). For any scoring function ***f:X → R***, the AUC represents the probability that a positive sample is ranked higher than a negative sample. It is defined as:


(6) \begin{equation*} \boldsymbol{AUC}\left(\boldsymbol{f}\right)=\boldsymbol{\Pr}\left(\boldsymbol{f}\left(\boldsymbol{x}\right)\ge \boldsymbol{f}\left({\boldsymbol{x}}^{\prime}\right)|\boldsymbol{y}=\mathbf{1},\boldsymbol{y}=-\mathbf{1}\right) \end{equation*}

where ***f(x)*** and ***f(x′)*** denote the scores assigned by the decision function, where ***x*** represents a positive sample (***y = 1***) and ***x′*** a negative sample (***y′ = −1***). The objective is to determine the optimal decision function ***f*** that maximizes this probability:


(7) \begin{eqnarray*}&& \boldsymbol{\arg}\ \underset{\boldsymbol{f}}{\boldsymbol{\max}}\boldsymbol{AUC}\left(\boldsymbol{f}\right) = \boldsymbol{\arg}\ \underset{\boldsymbol{f}}{\boldsymbol{\min}}\boldsymbol{\Pr}\left(\boldsymbol{f}\left(\boldsymbol{x}\right)<\boldsymbol{f}\left({\boldsymbol{x}}^{\prime}\right)|\boldsymbol{y}=\mathbf{1},\boldsymbol{y}=-\mathbf{1}\right)\nonumber\\ &&\quad\qquad =\, \boldsymbol{\arg}\ \underset{\boldsymbol{f}}{\boldsymbol{\min}}\ \mathbb{E}\left[{\boldsymbol{\pi}}_{\boldsymbol{f}\left({\boldsymbol{x}}^{\prime}\right)-\boldsymbol{f}\left(\boldsymbol{x}\right)>\mathbf{0}}|\boldsymbol{y}=\mathbf{1},\boldsymbol{y}=-\mathbf{1}\right] \end{eqnarray*}

If the parameter is true, the function $\boldsymbol{\pi} \left(\bullet \right)$ returns 1; otherwise, it returns 0. Due to its discontinuity, the function is typically approximated by a convex function. In this study, we use a loss function ${\mathbf{\mathcal{L}}}_{\mathbf{2}}\boldsymbol{loss}{\left(\mathbf{1}-\left(\boldsymbol{f}\left({\boldsymbol{x}}^{\prime}\right)-\boldsymbol{f}\left(\boldsymbol{x}\right)\right)\right)}^{\mathbf{2}}$ as the convex surrogate. Specifically, we define ***f(x)* = *w***^***T***^***x***, where ***w*** represents the parameters of the deep neural network to be learned. Consequently, the AUC- maximization problem can be formulated as the following optimization:


(8) \begin{equation*} \boldsymbol{\arg}\ {\boldsymbol{\min}}_{\left\Vert \boldsymbol{W}\right\Vert \le \boldsymbol{R}}\boldsymbol{E}\left[{\left(\mathbf{1}-{\boldsymbol{w}}^{\boldsymbol{T}}\left(\boldsymbol{x}-{\boldsymbol{x}}^{\prime}\right)\right)}^{\mathbf{2}}|\boldsymbol{y}=\mathbf{1},\boldsymbol{y}=-\mathbf{1}\right] \end{equation*}

Then this optimization problem can be reformulated as a stochastic saddle-point problem [28]. Accordingly, we define the function ***F: R***^***d***^ ***× R***^***3***^ ***× Z → R***, and AUC loss function (***L***_***AUC***_) as follows:


(9) \begin{eqnarray*}&& \mathbf{F}\left(\mathbf{w},\mathbf{a},\mathbf{b},\boldsymbol{\mathrm{\alpha}}; \mathbf{z}\right)=\left(\mathbf{1}-\mathbf{p}\right){\left({\mathbf{w}}^{\mathbf{T}}\mathbf{x}-\mathbf{a}\right)}^{\mathbf{2}}{\mathbf{I}}_{\left[\mathbf{y}=\mathbf{1}\right]}+\mathbf{p}{\left({\mathbf{w}}^{\mathbf{T}}\mathbf{x}-\mathbf{b}\right)}^{\mathbf{2}}{\mathbf{I}}_{\left[\mathbf{y}=-\mathbf{1}\right]}\nonumber\\&& \quad+\,\mathbf{2}\left(\mathbf{1}+\boldsymbol{\mathrm{\alpha}} \right)\left({\mathbf{pw}}^{\mathbf{T}}\mathbf{x}{\mathbf{I}}_{\left[\mathbf{y}=-\mathbf{1}\right]}-\left(\mathbf{1}-\mathbf{p}\right){\mathbf{w}}^{\mathbf{T}}\mathbf{x}{\mathbf{I}}_{\left[\mathbf{y}=\mathbf{1}\right]}\right)-\mathbf{p}\left(\mathbf{1}-\mathbf{p}\right){\boldsymbol{\mathrm{\alpha}}}^{\mathbf{2}} \end{eqnarray*}

where ***a*** and ***b*** represent the average predicted scores for positive and negative samples, respectively. The hyperparameter $\boldsymbol{\alpha}$ controls the trade-off between these scores. Additionally, ***p*** denotes the probability that a sample belongs to the positive class. The final loss function is defined as follows, where $\boldsymbol{\tau}$ is a hyperparameter that controls the weighting of different components.


(10) \begin{equation*} \boldsymbol{L}=\boldsymbol{\tau} \times{\boldsymbol{L}}_{\boldsymbol{AUC}}+\left(\mathbf{1}-\boldsymbol{\tau} \right)\times{\boldsymbol{L}}_{\boldsymbol{BCE}} \end{equation*}

The motivation for employing a mixture of AUC and binary cross-entropy (BCE) losses strategy is as follows. Although AUC loss is effective in addressing class imbalance by optimizing ranking performance, it exhibits weak probability calibration, particularly during the early training stages. In contrast, BCE loss, as a standard objective for probability calibration, offers more stable gradients and better discrimination. Thus, BCE loss serves as an auxiliary supervision term, while AUC loss focuses on optimizing the model’s ranking capability. In summary, the integration of the AUC-maximization strategy into the TEB specificity prediction task optimizes the model’s training process. This approach effectively mitigates the issue of data imbalance, improving both training efficiency and predictive accuracy.

## Results

This study first introduces the comparative model and outlines the experimental setup. It then evaluates and compares the performance of GRAPE with the comparative model using four public datasets. Ablation and parameter sensitivity experiments are subsequently performed to assess the contribution of each key module and the model’s responsiveness to parameter changes. Additionally, to evaluate the model’s generalization capability, further analysis is conducted on an independent test set. Lastly, a case study is presented, focusing on the detection of key contact sites within the TCR pool.

### Experimental setup

In TEB specificity prediction, five-fold cross-validation is commonly used to evaluate model performance [15–17]. Traditional methods often employ random data splitting, but this can lead to data leakage, particularly when identical TCR sequences appear in both training and validation sets. Such leakage may result in overly optimistic performance estimates, as the model encounters previously seen TCRs during training. To mitigate this issue, this study adopts the StrictTCR strategy proposed by Jiang *et al.* for data partitioning, followed by five-fold cross-validation [18]. This approach ensures consistent evaluation conditions across all models. Specifically, TCR sequences are grouped based on unique identifiers and divided into five independent subsets, preventing any TCR from appearing in both training and validation sets. Furthermore, during negative sample generation, StrictTCR eliminates overlap between negative samples in the training and validation sets. This segmentation strategy enhances the scientific rigor of the experimental setup, facilitating a more reliable assessment of the model’s generalization to unseen TCR data. Model performance is evaluated using two standard metrics: AUC and AUPR.

### Performance comparison

**Figure 2:**
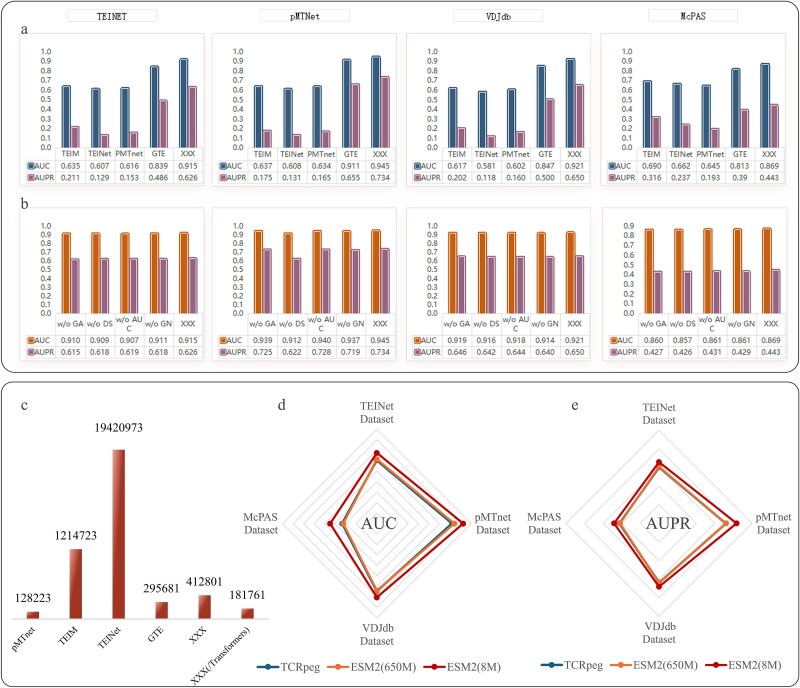
(a) The performance of GRAPE and the baseline model across four datasets, (b) results of ablation experiments across four datasets, (c) analysis of the model’s complexity, (d) AUC and (e) AUPR scores with different pretrained models.

We evaluated the performance of GRAPE and the baseline model across four datasets: TEINet [15], VDJdb [10], pMTNet [17], and McPAS [12]. Fig. 2a presents the results of five-fold cross-validation using the StrictTCR splitting method for all models on these datasets. Our model shows substantial improvements over the baseline, especially on the TEINet and VDJdb datasets. Specifically, we achieved a 7-percentage-point increase in AUC and a 15-percentage-point increase in AUPR, significantly outperforming existing benchmark models. Although the GTE model also incorporates topological structure information, it relies on embeddings generated by the TCRpeg method. TCRpeg is a pretrained model based on 106 TCR sequences and 362 456 unique epitopes, which limits its performance due to the small dataset size and lack of generalizability. In contrast, our approach uses ESM2.0, a large-scale protein language model, which greatly enhances the generalization and expressive power of the embeddings. These results validate the effectiveness of our method, demonstrating the advantages of combining a broadly applicable pretrained model (ESM2.0) with a dynamic topological architecture based on Transformers.

On the pMTNet dataset, the GRAPE method achieved the highest AUC score of 0.945 and an AUPR score of 0.734, significantly outperforming all baseline methods. This success is attributed to the effective construction of a high-quality TCR-epitope bipartite graph by the GRAPE method. Furthermore, the GRAPE method’s performance on pMTNet exceeds that on the other three datasets. This may be due to the pMTNet dataset, which combines data from multiple sources and undergoes rigorous screening to retain only high-confidence samples. In contrast, the other datasets may include lower-confidence samples, which could affect model performance. Despite this, the GRAPE method still significantly outperforms all baseline models on the three datasets that may contain noisy data. These results suggest that baseline methods struggle to adapt to diverse data sources, particularly exhibiting sensitivity to low-confidence data. In contrast, our dynamic topological structure not only captures inherent dataset differences but also demonstrates robustness to low-confidence data, further validating the superiority and broad applicability of the GRAPE method.

We also conducted a comprehensive analysis of the model’s complexity, as shown in Fig. 2c. The GRAPE model, utilizing the Transformer architecture with a global attention mechanism, maintains a relatively low parameter count, striking a good balance between predictive accuracy and computational efficiency. Notably, when the Transformer architecture is omitted, the GRAPE model’s parameter count drops to under 200 000, yet its performance still significantly outperforms the baseline model, GTE. For example, on the TEINet dataset, the GRAPE model leads by ~7 points in AUC and 13 points in AUPR. This highlights that the GRAPE model not only offers superior accuracy but also demonstrates high computational efficiency.

**Figure 3:**
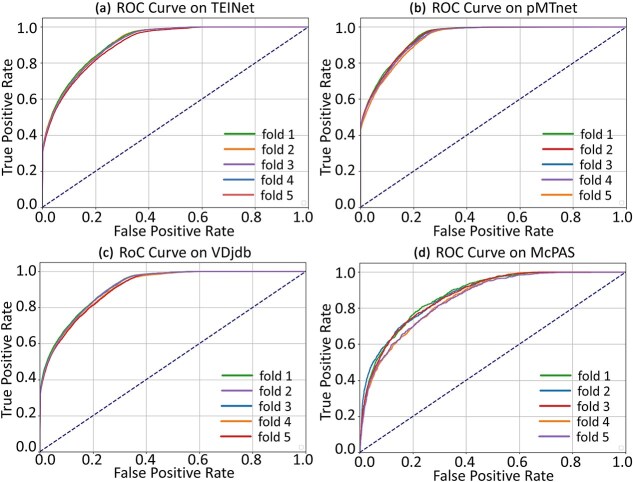
The ROC curves of GRAPE on (a) TEINet, (b) pMTnet, (c) VDjdb, and (d) McPAS datasets, respectively.

Additionally, evaluating model performance under low-density interaction scenarios is crucial, as a robust model should generalize not only to dense benchmark datasets but also to sparse real-world conditions. This study incorporated low-density interaction datasets in the validation experiments. For instance, as shown in Table 1, the McPAS dataset includes 5102 known TCR-epitope interactions involving 4975 TCRs and 244 epitopes, from which the interaction density ($5102\div \left(244\times 4975\right)\approx 0.0042$) can be derived. Similarly, interaction densities for the TEINet, pMTnet, and VDJdb datasets were also calculated ($44682\div \left(41610\times 180\right)\approx 0.0060$, $31203\div \left(28604\times 426\right)\approx 0.0027$, $38533\div \left(36033\times 1002\right)\approx 0.0011$). These datasets exhibit extremely low interaction densities, with average node degrees close to 1. We conducted five-fold cross-validation on these sparse datasets, and the corresponding AUC curves are presented in Fig. 3. The results demonstrate that the GRAPE model maintains competitive performance under sparse conditions, indicating its strong adaptability. This robustness may stem from the dynamic negative sampling strategy, which continuously injects previously unseen edges into the graph, mitigating overfitting and node isolation.

### Ablation experiment

This study conducted ablation experiments across four datasets to evaluate the contributions of key model components, with results summarized in Fig. 2b. The abbreviations “w/o GA,” “w/o DS,” “w/o AUC,” “w/o GN”, and “w/o ALL” denote models without the global attention mechanism, dynamic negative sampling, AUC loss component, graph regularization, and all components, respectively. The complete model is referred to as GRAPE. The results show that removing any single component—such as global attention, dynamic negative sampling, AUC loss, or graph regularization—results in only a slight performance drop. For example, on the pMTNet dataset, removing the global attention mechanism decreases the AUC from 0.945 to 0.939 and the AUPR from 0.734 to 0.725. However, removing all components leads to a substantial degradation in performance, especially on the pMTNet, VDJdb, and McPAS datasets. These findings suggest that the combined effect of all components is critical, while the individual contribution of each component is relatively limited. We will continue to investigate this phenomenon in future work.

### Pretrained model analysis

This study employs the ESM protein language model to extract initial representations of TCRs and epitopes, enabling the construction of a TCR-epitope bipartite graph. To evaluate the impact of different pretrained models on overall performance, we conducted a comparative analysis using the Transformer-based model, ESM2.0 and the RNN- and autoencoder-based model, and TCRpeg [29]. ESM2.0, a state-of-the-art biological language model, is designed for protein sequence analysis and functional prediction. Pretrained on large-scale biological sequence data, it effectively captures complex features and functional correlations within protein sequences, demonstrating superior performance across various bioinformatics tasks. In contrast, TCRpeg incorporates GRU layers to model relationships between TCR and epitope features. Trained on a specialized dataset containing 106 TCR sequences and 362 456 unique epitopes, TCRpeg generates pretrained embeddings. However, its generalization ability is limited by the relatively small size of the dataset.

This study evaluates the performance of three pre trained models-ESM2.0 (650 M and 8 M versions) and TCRpeg across four datasets. To ensure a fair comparison, all parameters of the GRAPE model and data inputs remained consistent, with only the pretrained models used to extract TCR and epitope sequence representations. Fig. 2d and e present the average AUC and AUPR results of five-fold cross-validation for the GRAPE model with different pretrained models, respectively. The results reveal substantial performance differences based on the choice of pretrained model. Specifically, the 8 M version of ESM2.0 achieves the highest performance, significantly outperforming the other models. However, when the parameter size of ESM2.0 increases to 650 M, its performance becomes comparable to TCRpeg. This may be attributed to the relatively short length of TCR and epitope sequences, which exhibit simpler interaction patterns. Consequently, a large parameter capacity is not necessarily required to capture these relationships. The 8 M version is better suited for tasks of medium to low complexity, effectively capturing key patterns in the data while maintaining model simplicity. In contrast, the larger model may attempt to fit more intricate patterns within the embeddings, but such overfitting does not necessarily enhance task performance. The smaller parameter model, by leveraging global information in the embeddings, demonstrates greater robustness.

In summary, two key insights emerge from these findings. First, utilizing large-scale protein language models to extract TCR and epitope representations significantly improves performance in tasks involving amino acid sequences. Second, aligning model capacity with task complexity is crucial for optimization. For short sequences such as TCRs and epitopes, smaller models can effectively leverage high-quality embeddings from large protein language models, achieving performance gains while reducing the risk of overfitting. These insights offer valuable guidance for future research on applying large-scale protein pretrained models to immunological tasks.

### Parameter sensitivity analysis

**Figure 4:**
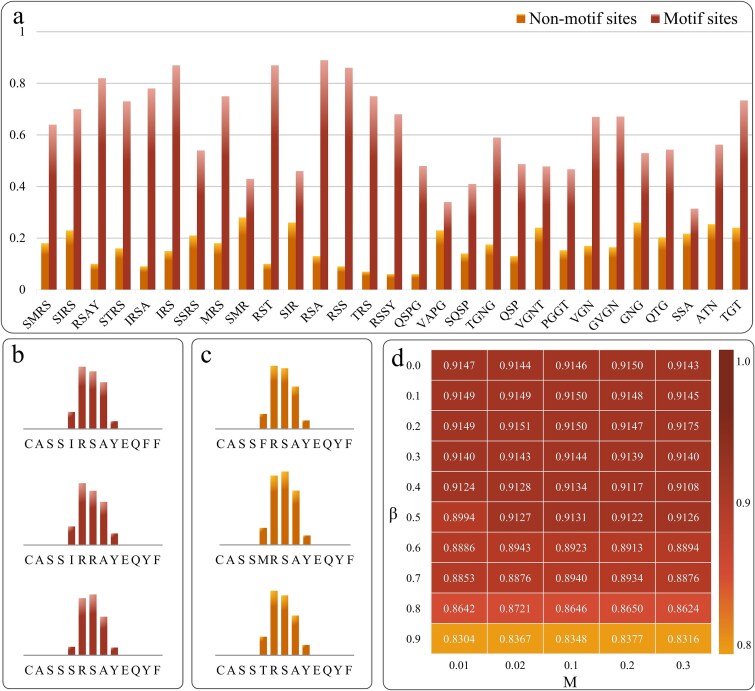
Analysis of the TCR pool for three specific epitopes. (a) Predicted contact scores for motif and nonmotif regions, presented as mean values with 95% confidence intervals. The x-axis represents motifs, while the y-axis represents contact scores; (b and c) predicted contact scores for six CDR3β sequences containing the RSAY motif and a single residue from the dataset; (d) GRAPE model performance heatmap under different combinations of $\boldsymbol{\tau}$ and M parameters ($\boldsymbol{\tau}$ represents the weight of the AUC loss, M represents the sampling probability for dynamic edge updates).

This study conducted a parameter sensitivity analysis on the McPAS dataset to assess the stability of the GRAPE model. The experiments followed a five-fold cross-validation approach, where four folds were used for training and one for testing. The analysis focused on two key parameters: $\boldsymbol{\tau}$, which determines the weight of the AUC loss, and ***M***, which controls the sampling probability for dynamic edge updates. $\boldsymbol{\tau}$ was varied from 0 to 0.9 in increments of 0.1, while ***M*** was tested at 0.01, 0.02, 0.1, 0.2, and 0.3. Keeping all other hyperparameters and input data constant, we evaluated 50 different parameter combinations, with results presented in Fig. 4d.

The findings indicate that the model remains robust to variations in $\boldsymbol{\tau}$ within the range of 0 to 0.7, maintaining stable performance. However, when $\boldsymbol{\tau}$ reaches one (eliminating the BCE loss), the AUC score declines significantly, underscoring the essential role of BCE loss as a balancing factor in the joint loss design. Similarly, model performance remains stable across the entire range of ***M*** values (0.01–0.3), further confirming its robustness. Overall, these results demonstrate that the GRAPE model maintains stable performance across a wide range of $\boldsymbol{\tau}$ and ***M*** values, highlighting its strong generalization ability and insensitivity to hyperparameter variations. This robustness enhances the model’s adaptability, offering flexibility in hyperparameter tuning for practical applications.

### Evaluation on the independent dataset

This study evaluated the performance of GRAPE and comparative models on an independent PDB test set, provided by the baseline model TEINet. The test set was derived from the RCSB Protein Data Bank (PDB) [30] and originally contained 105 TCR-epitope complex structures, which were screened down to 63 high-confidence positive samples. The PDB test set maintains a relatively balanced distribution, with each epitope binding to only one or two TCRs, presenting a unique challenge. To ensure a fair comparison, the TEINet dataset was used for training, and negative samples were generated at a 1:1 ratio using the StrictTCR strategy, applying the same negative sampling method to the PDB test set. All models underwent five-fold cross-validation to determine optimal configurations before evaluation on the independent test set. As shown in Table 2, the GRAPE model significantly outperforms all baseline models, demonstrating superior ability to capture complex TCR-epitope interactions while maintaining robustness and adaptability under isolated conditions.

**Table 2:** Results of all models on the independent dataset

Methods/metrics	AUC	AUPR
TEIM	0.586	0.573
TEINet	0.561	0.639
PMTnet	0.616	0.153
GTE	0.645	0.642
GRAPE	0.698	0.686

The AUC and AUPR scores of the proposed GRAPE model drop markedly on the independent test set, likely due to substantial distributional differences between the training and test sets, such as the presence of unique structural patterns or isolated conformations in the test data. These discrepancies lead to significant variations in the constructed TCR-epitope bipartite graph. In contrast, baseline models such as TEIM, TEINet, PMTnet, and GTE exhibit more stable performance. Specifically, TEIM, TEINet, and PMTnet rely solely on amino acid sequences and do not construct a TCR-epitope graph, making them less sensitive to distribution shifts. Although GTE also constructs a bipartite graph, GRAPE integrates dynamic negative sampling, global attention, deep AUC loss, and graph regularization, offering superior robustness under distribution shifts.

To further investigate this, we conducted ablation experiments. As shown in Table 3, removing any single component—global attention, dynamic negative sampling, AUC loss, or graph regularization—does not significantly impact the model’s degradation. However, removing all components leads to a substantial drop in performance, indicating that these modules function synergistically. Overall, the GRAPE model outperforms existing baselines but still requires improvement in generalization. In future work, we plan to leverage large-scale models and knowledge distillation techniques to extract TCR-epitope interaction patterns from broader protein–protein interaction datasets, thereby enhancing generalizability.

**Table 3:** Results of ablation experiments on the independent test set

Conditions/metrics	AUC	AUPR
w/o GA	0.694	0.681
w/o DS	0.688	0.676
w/o AUC	0.693	0.679
w/o GN	0.694	0.678
w/o ALL	0.665	0.649
GRAPE	0.698	0.686

Furthermore, this study conducted multiple case analyses to evaluate the GRAPE model’s ability to detect TEB sites. Among various TCRs targeting specific epitopes, the GRAPE model accurately predicts TEB conformations, identifying key contact sites essential for understanding TCR-epitope interactions. This insight is particularly valuable for elucidating binding mechanisms. Notably, TCR-epitope interactions are more likely to occur within motif regions. Glanville *et al.* proposed the GLIPH method to identify enriched CDR3β motifs within TCR repertoires [19]. To validate the effectiveness of GRAPE, we compared its predicted contact scores with the CDR3β motifs identified by GLIPH. Additionally, we analyzed differences in contact scores between motif and nonmotif regions to further assess the model’s predictive accuracy.

As shown in Fig. 4a, the predicted contact scores for motif regions identified by GLIPH are significantly higher than those for nonmotif regions. Fig. 4b and c present six representative cases containing the RSAY motif, where the predicted contact scores for these CDR3β sequences are markedly higher than those of nonmotif regions. These findings confirm the GRAPE model’s ability to accurately capture key contact sites involved in epitope binding.

## Discussion

Early TEB specificity prediction models relied on large epitope-specific TCR datasets, which are often difficult to obtain. To address this limitation, more generalized models, such as pMTnet, TEINet, and GTE, have been developed, demonstrating potential in cancer progression analysis, prognosis evaluation, and immunotherapy response prediction. However, the short length of TCR and epitope sequences poses challenges for the application of Multiple Sequence Alignment (MSA)-based protein models. While single-sequence protein language models like ESM2.0 offer potential, their direct integration into previous models has not yielded optimal results. Recent advances in GNNs have shown promise in TEB prediction but still encounter challenges such as data scarcity and over-smoothing. To address these issues, this study proposes a novel model that integrates protein language models with graph regularization techniques, enhancing the understanding of TEB mechanisms while effectively capturing long-range dependencies and mitigating challenges associated with sparse node representations.

The GRAPE model significantly outperformed state-of-the-art methods across four public datasets. Notably, on the TEINet and VDJdb datasets, the AUC improved by ~7 percentage points, while the AUPR increased by ~15 percentage points. Ablation experiments on these datasets demonstrated that key components, including the global attention mechanism, dynamic negative sampling strategy, and AUC loss, were essential for enhancing model performance. Furthermore, a parameter sensitivity analysis on the McPAS dataset revealed that the model is highly robust to variations in the AUC loss weight and maintains stable performance across a wide range of parameter settings.

Similar to most existing methods, the GRAPE model primarily utilizes information from the CDR3 region of the TCR β-chain for modeling. However, previous research suggests that relying solely on CDR3β may limit predictive performance due to the relatively low quality and limited information content of these data. In contrast, incorporating paired information from both the TCR α-chain and β-chain provides a more comprehensive feature representation [31, 32]. However, only a subset of entries in major databases (e.g. VDJdb and McPAS) include α–β pairing information. To ensure model consistency and scalability across datasets, this study focuses on the β chain [33]. To address this limitation, future work will focus on integrating full-chain TCR information by designing a more advanced heterogeneous GNN. This enhancement will enable the model to better capture and utilize these additional features, thereby improving predictive accuracy and generalization. Furthermore, the GRAPE model has already demonstrated significant improvements in TEB specificity prediction, highlighting the effectiveness of large-scale pretrained protein language models. This approach offers new insights into overcoming challenges associated with short TCR and epitope sequences in sparse datasets. Given its integration of dynamic graph structures and Transformer architectures, the GRAPE model exhibits relatively high computational complexity while effectively capturing intricate interaction relationships. Future research will explore strategies such as incorporating additional pr trained models and optimizing computational efficiency through techniques like model pruning, knowledge distillation, and quantization. These advancements will help accommodate large-scale datasets while maintaining robust predictive performance.

## Conclusion

TCRs activate immune responses by recognizing epitopes presented by MHC molecules, playing a crucial role in eliminating tumor cells and pathogens. Understanding TEB mechanisms is essential for cancer immunology, autoimmune antigen discovery, and vaccine design. However, the complexity and high cost of experimentally validating these interactions underscore the need for effective computational approaches. Existing GNN-based methods encounter challenges in capturing long-range dependencies and often suffer from over-smoothing during message propagation due to sparse nodes, limiting their ability to generate high-quality initial representations. Additionally, class imbalance in TCR-epitope datasets further complicates predictive modeling. To address these challenges, this study introduces a TEB specificity prediction model that integrates protein language models with graph regularization techniques. First, protein language models generate enriched representations of TCRs and epitopes, forming a more comprehensive TCR-epitope bipartite graph. Next, an encoder incorporating graph regularization and global attention mechanisms is designed to capture both local graph features and global dependencies. This encoder extracts robust representations while mitigating over-smoothing effects. Furthermore, dynamic graph learning strategies and AUC-maximization techniques are employed to handle class imbalance. The dynamic learning strategy enhances model training efficiency and predictive accuracy by adaptively updating negative TCR-epitope pairs.

**Key Points**

- GRAPE: TEB predictor fuses protein language model & graph regularization, capturing local/global features with reduced over-smoothing.
- Dynamic graph learning & AUC max tackle data imbalance, ensuring robustness.
- GRAPE achieves SOTA in benchmarks, boosting therapeutic discovery in cancer immunology and vaccine design.

## Data Availability

Our code and data will be publicly available at: https://github.com/Excelsior511/GRAPE/.
